# Letter to the editor: “Eliciting national and subnational sets of disability weights in mainland China: findings from the Chinese disability weight measurement study”

**DOI:** 10.1016/j.lanwpc.2024.101008

**Published:** 2024-01-17

**Authors:** Thomas Hampton, Kathryn A. Haigh, Mphatso Dennis Phiri, Ewan Tomeny

**Affiliations:** a University of Liverpool, Liverpool, UK; b Liverpool School of Tropical Medicine, Liverpool, UK; c Malawi Liverpool Wellcome Research Programme, Blantyre, Malawi; d Centre for Infectious Diseases Research in Africa, University of Cape Town, South Africa

We commend Liu et al. for providing new disability weights (DW) based on 468,541 respondents from across mainland China following Global Burden of Disease (GBD) study methodology.^1^ A low proportion of respondents from China contributed to prior GBD surveys and similar studies from Japan found differences compared to the GBD DW values.^2^ However, some reported weights seem to defy condition severity classifications, contradicting conventional clinical and health economic expectations.

For the five ordinal states reflecting severity of hearing loss (HL), despite a clear worsening of lay descriptions, state values provided imply a benefit to complete HL over profound or severe HL (Table 1; Fig. 1). Furthermore, the weights for Tuberculosis (TB) suggest a lower disability level for individuals with TB who are additionally living with HIV. While arguments could be invoked for valid differences in care/experiences in these groups such as adjustment in social or occupational contexts, signposting, care delivery models, follow-up frequency etc,^3^ this cannot explain these incongruous DWs, due to worsening lay descriptions used in the weight elicitation. These DW were established by presenting participants with a selection of lay descriptions (not the whole batch or the disease state names), in a process of pairwise comparison. Health states themselves were not given, and ultimate weights were estimated using statistical modelling.^1^

**Table 1:** DW from Table 2 in Liu et al.^1^ (The DW is measured on a scale from 0 to 1, with a score of 0 indicating a state equivalent to full health and score of 1, essentially a state equivalent to death).

Disability weight (95% UI)			Lay descriptions
**Tuberculosis**			
16	Not HIV infected	0.375 (0.319–0.435)	has a persistent cough and fever, is short of breath, feels weak, and has lost a lot of weight.
17	HIV infected	0.297 (0.216–0.390)	has a persistent cough and fever, shortness of breath, night sweats, weakness and **fatigue** and **severe** weight loss.
**Hearing loss**			
105	Mild	0.031 (0.003–0.122)	has great difficulty hearing and understanding another person talking in a noisy place (for example, on an urban street).
106	Moderate	0.068 (0.014–0.192)	is unable to hear and understand another person talking in a noisy place (for example, on an urban street), and has difficulty hearing another person talking even in a quiet place or on the phone.
107	Severe	0.246 (0.156–0.358)	is unable to hear and understand another person talking, even in a quiet place, and unable to take part in a phone conversation. Difficulties with communicating and relating to others cause emotional impact at times (for example worry or depression).
108	Profound	0.200 (0.109–0.327)	is unable to hear and understand another person talking, even in a quiet place, is unable to take part in a phone conversation, and has great difficulty hearing anything in any other situation. Difficulties with communicating and relating to others often cause worry, depression, and loneliness.
109	Complete	0.151 (0.065–0.287)	cannot hear at all in any situation, including even the loudest sounds, and cannot communicate verbally or use a phone. Difficulties with communicating and relating to others often cause worry, depression or loneliness.

**Fig. 1:**
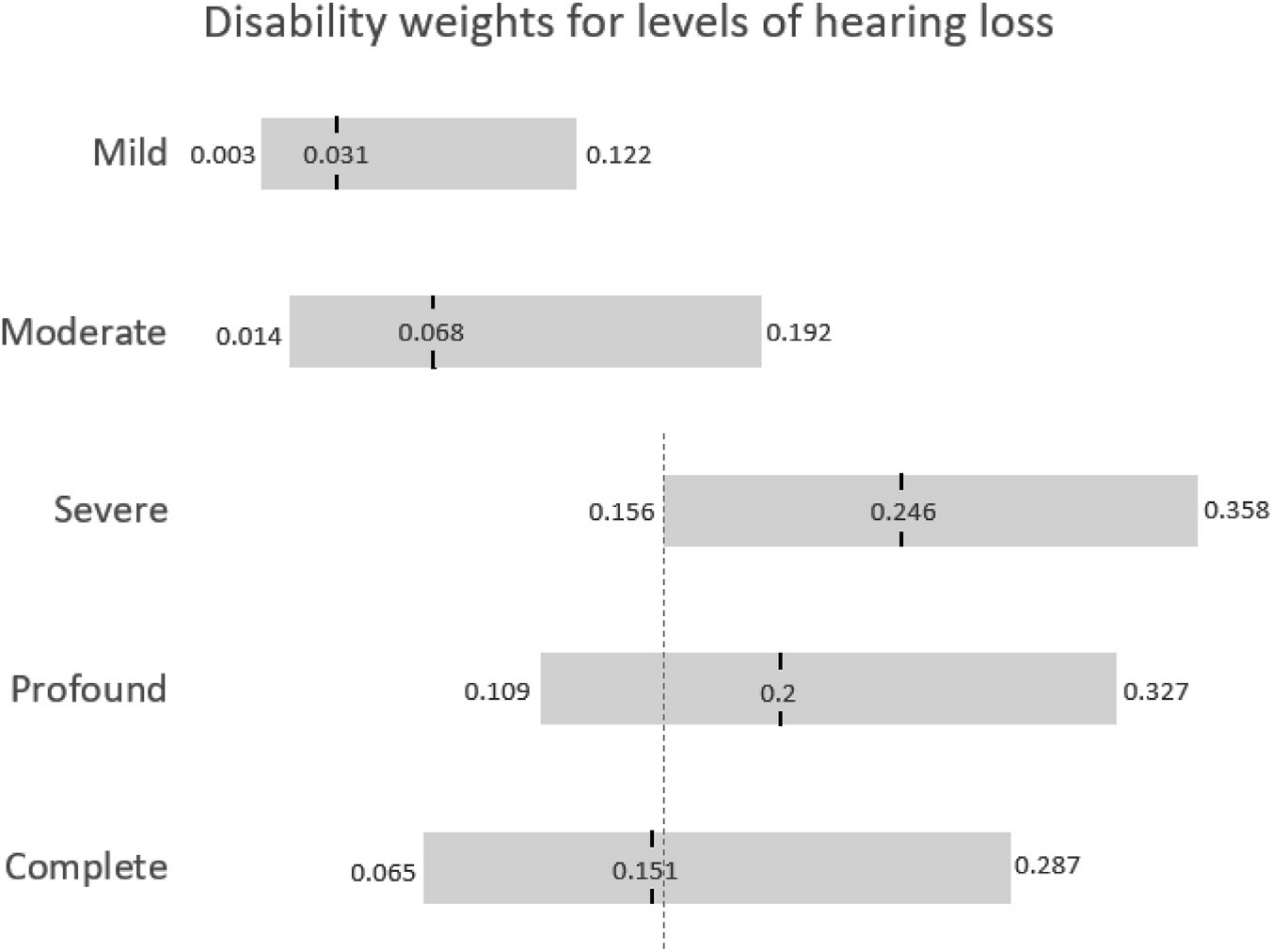
DW point estimates from Table 2 in Liu et al.^1^ (There is considerable overlap in 95% uncertainty intervals which could yet contain the true estimate but the DW point estimates themselves suggest a clear improvement in health state despite lay descriptions worsening [in particular, DW estimates for profound and complete HL suggest lower severity compared to the severe HL state (blue dashed line indicates lower bound of severe HL)].

From a health economic or policy position, these values are concerning, potentially leading to inappropriate conclusions. While weights are presented with uncertainty intervals, researchers often rely on point estimates. These DW values imply there would be no benefit in restoring the hearing of people with complete HL to that of an individual with severe HL (even if this could be achieved at no cost). Models using these weights could concerningly conclude that to reduce disability in a given population, an appropriate intervention might be to *remove* remaining hearing from some members or, for TB, suggest that coinfection with HIV is beneficial.

Others have previously expressed wider concerns regarding GBD weight derivation methodology^4^ and these contradictory findings may affect confidence in values assigned to other conditions, particularly across health states less easily verified than those above with well-defined severity levels. It seems necessary that future weight derivation studies sense-check for such reversals with participants, the public and/or clinicians, and we caution against the publication of weight tables containing incongruous results. These findings again raise the inherent issues that arise from using simple sentences as surrogates for complex conditions and applying a single set of DW across diverse global populations.^5^ Specifically, these verifiable concerns require addressing or risk worsening of health equity for populations with these conditions.

## Contributors

ET and TH conceptualised the article. TH wrote the original draft of the article. ET, MDP and KAH provided additional original writing. All authors contributed equally to critical appraisal, review & editing. All authors approved final submitted version.

## Declaration of interests

TH and KAH are supported by a Wellcome Trust Fellowship (203919/Z/16/Z). MDP is supported by a UK FCDO grant, OJEU reference number: 2018/S 196-44348. ET is supported by an institutional grant from UK aid: LIGHT consortium. No authors have any other potential or actual competing interests, financial or otherwise.
